# Dynamic Characterization of the CT Angiographic ‘Spot Sign’

**DOI:** 10.1371/journal.pone.0090431

**Published:** 2014-03-03

**Authors:** Santanu Chakraborty, Mohammed Alhazzaa, Jason K. Wasserman, Yang Yang Sun, Grant Stotts, Mathew J. Hogan, Andrew Demchuk, Richard I. Aviv, Dar Dowlatshahi

**Affiliations:** 1 Division of Neuroradiology, Department of Medical Imaging, Ottawa Hospital Research Institute and University of Ottawa, Ontario, Canada; 2 Department of Medicine, Ottawa Hospital Research Institute and University of Ottawa, Ontario, Canada; 3 Department of Epidemiology and Community Medicine, Ottawa Hospital Research Institute and University of Ottawa, Ontario, Canada; 4 Department of Pathology, Ottawa Hospital Research Institute and University of Ottawa, Ontario, Canada; 5 Department of Neurology, King Fahad Medical City, Riyadh, Kingdom of Saudi Arabia; 6 Division of Neuroradiology and Department of Medical Imaging, Sunnybrook Health Sciences Centre, University of Toronto, Toronto, Ontario, Canada; 7 Department of Clinical Neurosciences, Foothills Medical Centre, Calgary Health Region, Calgary, Alberta, Canada; INSERM U894, Centre de Psychiatrie et Neurosciences, Hopital Sainte-Anne and Université Paris 5, France

## Abstract

**Background and purpose:**

Standard (static) CT angiography is used to identify the intracerebral hemorrhage (ICH) spot sign. We used dynamic CT-angiography to describe spot sign characteristics and measurement parameters over 60-seconds of image acquisition.

**Methods:**

We prospectively identified consecutive patients presenting with acute ICH within 4.5 hours of symptom onset, and collected whole brain dynamic CT-angiography (dCTA). Spot parameters (earliest appearance, duration, maximum Hounsfield unit (HU), time to maximum HU, time to spot diagnostic definition, spot volume and hematoma volumes) were measured using volumetric analysis software.

**Result:**

We enrolled 34 patients: three were excluded due to secondary causes of ICH. Of the remaining 31 patients there were 18 females (58%) with median age 70 (range 47–86) and baseline hematoma volume 33 ml (range 0.7–103 ml). Positive dCTA spot sign was present in 13 patients (42%) visualized as an expanding 3-dimensional structure temporally evolving its morphology over the scan period. Median time to spot appearance was 21 s (range 15–35 seconds). This method allowed tracking of spots evolution until the end of venous phase (active extravasation) with median duration of 39 s (range 25–45 seconds). The average density and time to maximum density was 204HU and 30.8 s (range 23–31 s) respectively. Median time to spot diagnosis was 20.8 s using either 100 or 120HU definitions.

**Conclusion:**

Dynamic CTA allows a 3-dimensional assessment of spot sign formation during acute ICH, and captured higher spot sign prevalence than previously reported. This is the first study to describe and quantify spot sign characteristics using dCTA; these can be used in ongoing and upcoming ICH studies.

## Introduction

Computed Tomographic angiography (CTA) is non-invasive test that visualizes the transit of radio-opaque contrast through the vasculature, and is commonly used in acute cerebrovascular disease to identify sites of vessel occlusion or rupture. The CTA “spot sign” is a site of active contrast extravasation [1] during acute intracerebral hemorrhage (ICH) and an independent predictor of hematoma expansion and poor clinical outcomes. Although the majority of patients with ICH will experience hematoma expansion, the spot sign is only present in about a third [2], [3], [4], [5], [6]. This may be related to the use of routine “static” early phase CTA [3], [7] which necessitates pre-selection of image acquisition time at a set time-point following contrast injection; presently, the optimal standard CTA timing for spot visualization is unknown.

A recent evaluation of spot signs on delayed angiographic phases suggests its prevalence has been underestimated and confirmed the potential for delayed imaging to detect late phase spot signs [6], [8]. Accordingly, several techniques have been proposed to detect late phase spot signs such as post-contrast CT, CT-perfusion and dynamic CTA imaging [7], [8], [9], [10], [11], [12]. Longer image acquisition techniques capture up to 26% of late phase spot signs [10] and increases prevalence to 50% [9].

Dynamic CTA (dCTA) is a non-invasive low-radiation dose technique that acquires a time series of bone subtracted CTA images of the whole brain, which avoids the timing restrictions inherent to routine static CTA acquisition. The temporal wash-in and washout of intravascular contrast material over the scan duration provides the opportunity to assess for spot sign formation over the entire image acquisition protocol (typically 60 seconds), and to characterize its temporal evolution in a quantitative manner. We have previously used dCTA to explore the underlying pathology of the spot sign [1]. In the current study, we use dCTA to propose, define and describe dynamic spot sign characteristics that can be used in ongoing and upcoming ICH studies.

## Methods

### Study Design and Patient Population

Ottawa Health Science Network Research Ethics Board (OHSN-REB) approved this study with a waiver of consent for data collection. The Ottawa Hospital is a tertiary care stroke centre with a “code stroke” imaging protocol including non-contrast computerized tomography (NCCT), CTA of the neck arteries, and whole brain CT-perfusion. All patients presenting less than 4.5 hours with symptoms consistent with stroke or ICH and no known contraindications to iodinated contrast undergo the full imaging protocol as part of clinical care. We prospectively identified consecutive patients presenting with acute non-traumatic ICH, and collected imaging data performed as part of the “code stroke” protocol.

### Image Acquisition

**Figure 1:**
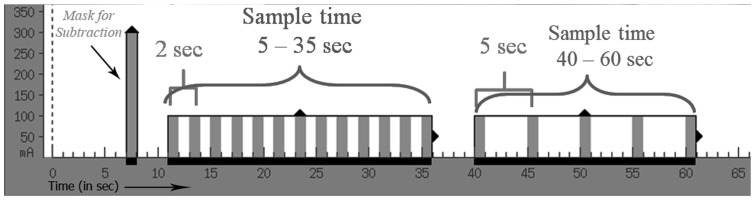
The dCTA image acquisition protocol: The timing line (x-axis in seconds) explains the acquisition of whole brain volumes at sequential time points starting at 7 sec from the time of contrast injection. This volume uses 300(10 to 35 sec), whole brain volumes are acquired every 2 sec. In the venous phase (40 to 60 sec) volumes are acquired at 5 sec interval.

We obtained baseline head CT, CTP and dynamic CTA images using Toshiba volume CT scanner (Aquilion ONE^TM^) as per our institutional stroke protocol. This system utilizes 320 ultra-high resolution detector rows (0.5 mm in width) to image the entire brain in a single gantry rotation, resulting in ultrafast scan times and reduced contrast and radiation exposures. We injected 40 ml of intravenous contrast at 5 ml/s through a power injector (Medrad® Stellant® CT Injection Systems) followed by a saline bolus. We acquired multiple low-dose (80 kVp) volume scans of the entire brain (skull base to vertex) in sequential order during contrast infusion to obtain whole brain perfusion and dynamic vascular analysis in one examination [13]. The data acquisition protocol and parameters are described in Figure 1. Image acquisition started at 7 seconds after contrast injection to obtain a mask for subtraction, and each volume took 1 second to acquire and consisted of 320 slices of 0.5 mm thickness to cover 16 cm along z-axis. Individual volumes were assessed as a standard CTA volume for that time point, and by combining all volumes together; we evaluated the passage of contrast through the intra and extra cranial circulation during the whole scanning protocol (60 seconds). The radiation dose per study was low with a DLP (Dose-Length-Product) of 1925 mGy-cm compared to a helical CT head scan (1349 mGy-cm). All scans were anonymized prior to analysis (by J.K.W.) using OsiriX (Pixmeo, Geneva).

### Imaging Analysis/Interpretation

An experienced neuroradiologist (S.C. 9 years' experience) reconstructed dynamic CTA data from the whole brain perfusion data into non-subtracted orthogonal multiplanar reformats, bone subtracted 3D MIP and volume rendered images using a Vitrea^TM^ workstation. Real-time contrast flow videos were generated, facilitating analysis of spot sign presence and its 3-dimensional features. We used Quantomo Pro (Cybertrial inc, Calgary, AB) [14], [15] to measure spot sign volumes and density. We calculated the spot sign parameters of earliest appearance, spot duration, maximum Hounsfield unit (HU), time to maximum HU, time to spot sign diagnostic criteria (based on HU>100 or >120) [5], [7], and the linear rate of spot sign formation (the change in volume of spot sign between its first visualization and the phase where it has reached the maximum density divided by time). An experienced stroke neurologist (D.D.) independently measured hematoma volumes, blind to spot status. Since the aim of this article is to describe and quantify the dynamic nature of spot sign, we did not provide predictive or direct comparative analysis between positive and negative spot groups.

## Results

**Figure 2:**
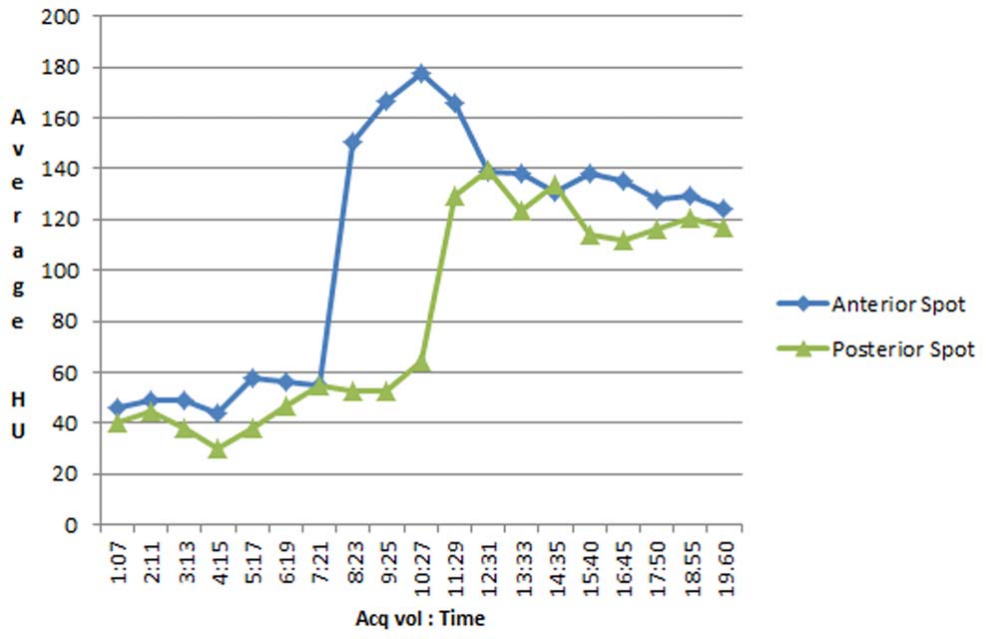
The graph shows the average contrast density (HU) over time for 2 spots in the same hematoma. The posterior spot appears approximately 3

Between December 2008 and June 2013, 34 consecutive patients presented with acute ICH were recruited who underwent dCTA as part of a code stroke protocol. Three patients with secondary ICH (underlying aneurysm, arteriovenous malformation and mycotic vasculitis) were excluded. Thirty-one patients with spontaneous ICH were included: 18 female (58%); median age 70 (range 47–86). The overall baseline hematoma volume was 33 ml (range 0.7–103 ml). Thirteen patients (42%) were dCTA spot sign (dspot) positive. Median baseline total hematoma volume in dspot positive patients was 48.6 ml (range 4–103) and dspot negative patients was 22 ml (range 0.7–94.1 ml) (p = 0.007). The median rate of contrast extravasation was 0.06 ml/s (range 0.04–1.1 ml/s). All dspots appeared in the arterial phase. In one case, dCTA showed a posteriorly located spot that appeared 3 seconds after an anterior one within the same hematoma (Figure 2, Video S1 & S2). One patient presented twice with different hemorrhages with a spot sign manifesting on the second occurrence. Multiple spots were noted in 3 hematomas.

### Dynamic spot sign characteristics

**Figure 3:**
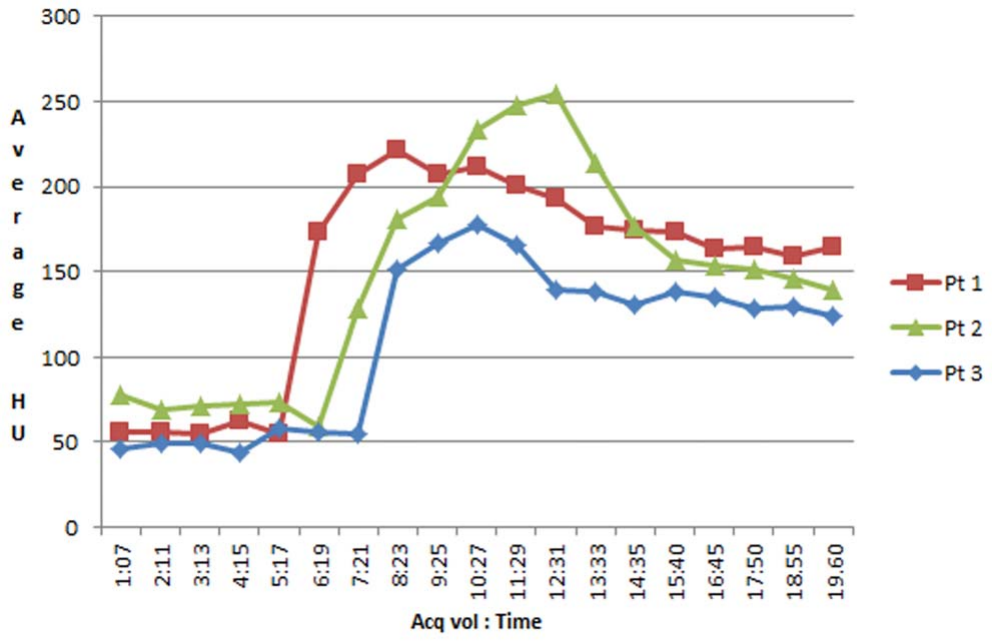
The graph demonstrates the average contrast density (HU) over time for spots in 3 representative patients. The average densities of the spots are diminishing before taking a plateau course toward the end of scanning, and remaining above the baseline.

**Figure 4:**
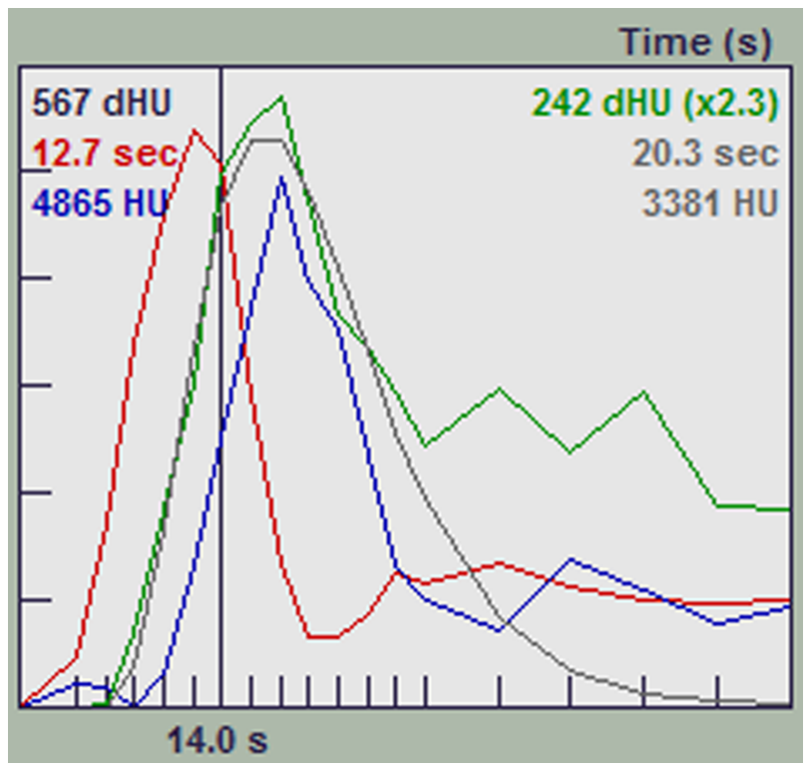
The graph shows the relation of point estimation of contrast density in Middle Cerebral Artery (in red), Superior Sagittal Sinus (in blue) and the spot sign (in green) in one patient. The spot sign density persists at a higher level than the baseline at the end of scanning duration.

**Figure 5:**
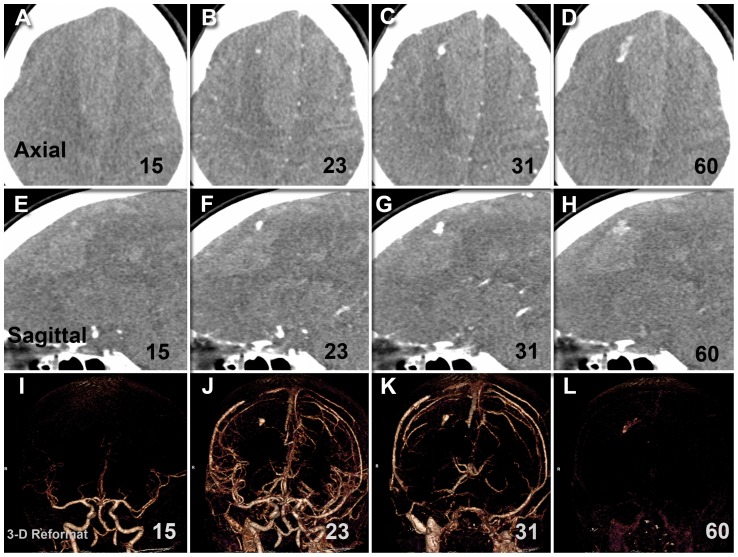
Dynamic CT angiographic image (Axial, sagittal, 3-D reformat) demonstrates the development of a spot sign over the arterial, mid-cycle and venous phases in a subject with large right parasagittal frontal hematoma. It is notable how the spot temporally evolved in its size, shape, volume and density and then eventually faded at the end of venous phase.

All spot signs continued through both arterial and venous phases (holophasic) until the end of image acquisition at 60 seconds. This continuity of spot sign presence over the whole scanning period allowed the assessment of contrast leakage between start and end points. The dspot was best appreciated on orthogonal non-subtracted multiplanar reformats when displayed as a continuous loop (video S1) [12], [16]. Such spots appeared as expanding 3-dimensional structures that evolved dynamically but variably in size, volume, shape, duration and sharpness of margin over the scan period. Variability was also noted in the dspot time-to-appearance and rate of expansion. Median time-to-spot sign diagnosis was 20.8 s, irrespective of the HU threshold used for spot sign definition (>100 or >120). To compare with static “first pass” arterial weighted CTA techniques that acquire images between 20–25 seconds following contrast bolus, our 42% spot sign rate would have been 35% at a 25-second acquisition, and only 19% at a 20-second acquisition. Dynamic spot signs appeared to spread along the surface or boundary of the hematoma. As the spots progressed in expansion, spots margins became indistinct and spots started losing their average density. In three cases, this resulted in fluctuation of objective spot volume measurement in later phases, as the semi-automatic measurement software uses threshold densities to identify spot margins. Average spot sign volumes started to decrease, but remained elevated (more than 10 fold) at the end of venous phase when compared to baseline at time of spot onset in the early arterial phase. The graph in Figure 3 shows the average contrast density in 3 representative spot signs, with baseline measurements of background hematoma density in the expected area of the images prior to the spot signs' appearance. The average density of the spot signs reduced due to dilution of contrast, but did not return to baseline (Figures 3, 4 and 5). Further features and parameters of dspot are shown in Table 1.

**Table 1:** Measurement parameters of dspot.

Characteristics	N = 13
Baseline hematoma volume, ml	48.6 (4–103)
Time from onset to CTA, min	115 (39–599)
Time to spot appearance, seconds	20.8 (15–35)
Duration of dSpot (leak), seconds	39 (25–45)
Maximum average density, HU	204
Time to reach Max. Density, seconds	30.8
Max. Spot volume (median), ml	0.43
Time from injection to max. Spot volume, seconds	46.6 (23–60)
Rate of contrast extravasation, ml/s	0.06
Spot volume, ml Early phase (at the onset of spot) Late phase (at 60s)	0.032 (0.001–0.12) 0.37 (0.001–2.8)

Median (range) listed for all continuous variables.

## Discussion

This study provides further insight into the dynamic evolution of the spot sign during acute primary ICH. In our series, dspot was present in 42% of patients, and contrast extravasation appeared to persist throughout the scanning period, with a median peak density of 204 HU at a median time-to-maximal density of 30.8 seconds. Moreover, dCTA allowed the assessment of rate of contrast extravasation and the measurement of several time and density parameters that may prove to be useful radiological surrogates of hematoma expansion in future ICH studies. Finally, dCTA facilitated the identification of spot signs by allowing us to easily visualize their contrast pooling in later images. Had our study relied on static first-pass arterial weighted CTA, our spot sign rate would have been 35% at 25 seconds post-contrast bolus, and only 19% at 20 seconds post-contrast bolus.

Choosing the optimal timing parameters for standard (static) CTA acquisition for detection of contrast leak and assessment of the spot sign is challenging [9]. Although the technical parameters of contrast injection rate, dose and volume can be standardized, physiological variables like circulation time, arterial resistance, and peri-hematoma/intracranial pressure will be different between patients and may influence the time to spot visualization. A single optimal CTA time for spot sign delineation for every patient may not be realistic. Dynamic CTA offers a solution with comparable contrast and radiation exposure as a standard static CTA.

Spot sign is not simply a dot in a hematoma as captured in standard CTA snapshot images; rather, it is a temporally evolving phenomenon [6]. Our study showed that dspot appeared as a holophasic (continuous in arterial and venous) evolving pool of contrast within the hematoma, reflecting the dynamic nature of acute ICH. We noticed that dspot volumes and densities have a crescendo pattern through the early and middle scanning phases, then decrease at the end of the venous phase, but do not return to baseline. We postulate this is due to dispersion, recirculation and dilution of contrast. However, it remains to be determined if spot volume, spot density, or estimates of the rate of leakage is predictive of hematoma expansion and clinical outcome; the predictive performance of these parameters must be tested in a larger sample size.

Our observed variability of extravasation rate, volumes, density and duration among our spot cases may reflect internal and external factors influencing dspot formation or “rate of leak”. Such factors are not yet well defined, but could relate to point-to-point physiological changes happening intracranially and systemically (such as blood pressure fluctuations, coagulation status and cardiac output), hematoma characteristics (such as underlying etiology, near ventricle location, and coagulation status) and radiographic factors (such as slice thickness, visualization window, contrast volume, and acquisition time after contrast injection).

### The real advantage of dCTA: the concept of “looking back”

The dCTA series gives us the opportunity not only to visualize the evolution and spreading of the spot over time but also the luxury of looking backwards. An obvious area of contrast pooling in the later images could be tracked back to its point of origin in earlier phases, which helped us to define confidently the presence, number and location of the spot in all cases. Our experience is consistent with studies that showed increased sensitivity and specificity of detecting spot sign and predicting of hematoma expansion by adding delayed imaging techniques including CTP source images [9] [10], delayed CTA [7] or postcontrast CT [8].

### Permeability versus dynamic spot: the “oil spill” effect

In contrast to abnormal permeability (e.g. tumor perfusion) where contrast leaks from abnormal vasculature in every voxel throughout the tumor, we found dspots all had a point source and expanded 3-dimensionally through the hematoma. This may be compared to an “oil spill” effect where the leakage has a point source followed by multidirectional expansion. In this scenario, the use of color perfusion maps that average out the contrast flow data may not be appropriate to estimate the rate of contrast extravasation in acute ICH.

Our study has several limitations. To limit the level of radiation exposure, we acquired low dose (80 kV) images which decreased the signal to noise ratio and potentially introduced more HU measurement errors as compared to standard 120 kV images; HU measurements were not corrected for this decreased dose. For similar reasons, we limited the temporal resolution to 2 seconds during the arterial phase and 5 seconds during the venous phase, which limited the assessment of spot sign flow dynamics. In addition, the patients within our study presented with large baseline hematoma volumes which may have contributed to the relatively high dynamic spot positive rate. Finally, this is a descriptive study describing dynamic spot sign characteristics and proposing measurable spot sign parameters, and was not intended to predict hematoma growth or clinical outcomes. The utility of our proposed spot sign parameters must be tested in a larger sample size, and a prospective study is in progress.

### Conclusion

In this pilot study, we describe dspot formation and propose quantifiable dspot parameters for future study. We further suggest that dCTA can facilitate the detection of spot signs using comparable contrast and radiation exposures to standard CTA. This technique can potentially improve imaging-based patient selection for emerging ICH therapies.

## Supporting Information

Video S1 **Sagittal reformatted images show temporal evolution of anterior and posterior spots in the same hematoma.**
(AVI)

Video S2 **Dynamic 3D MIP bone-subtracted images show temporal evolution of anterior and posterior spots in the same hematoma in a cut out volume.**
(AVI)
